# The Local Government Board Draft Nursing Order

**Published:** 1913-08-23

**Authors:** Jane Wilson

**Affiliations:** Honorary Treasurer, Workhouse Nursing Association.


### THE LOCAL GOVERNMENT BOARD DRAFT NURSING ORDER.

By MISS JANE WILSON, Honorary Treasurer, Workhouse Nursing Association.

I have been asked to give my opinion on this very important Draft Order. I do so willingly for The Hospital, which has during the last few years endeavoured to direct a constructive policy not only on nursing questions but on the whole vast idea of unity in treating the working-class sick by outdoor, hospital, or the Poor-Law systems. This admittedly is clearly essential and will arrive in due course—to stay. The effects of the Insurance Act have yet to be watched for several years before any real basis as to its effect on the ailing and sick can be arrived at. This being the case, all sorts of piecemeal scraps of attempts will continue to be seriously considered. We need not be too anxious therefore in points which seem retrograde in the new Draft Nursing Order. Nor is it needful at this date to consider the formation of the Committee of four gentlemen who have drawn up the Order.

The Interim Report.

With one exception the complete number—four—were *within* the Local Government Board, the exception being a barrister and clerk to the West Ham Guardians. That medical advice and inspectors should have been included on the Committee seems futile to mention now. We have the Order, and I will proceed to say where I think there is gain and where loss in comparison with a condition before 1897 and its Nursing Order. The Interim Report to Mr. Burns of a Draft Order, we understand, was not meant for circulation or publication, although extracts were made fully from it in the *Times* and *Poor-Law Officers' Journal* in September 1912. In any case it will be simpler to refer to the fact that the creation of superintendent nurses was a marked advance in the Order of 1897. It was the first recognition by the Local Government Board *by Order* that nurses existed as a trained class, that they were different from the persons who in 1854 "should be able to read and to write." Much good work was done by the creation of well-trained officials, with status and removal fixed, whose work was not terminable by Boards of Guardians only, but whose dismissal was impossible excepting with the approval of the Local Government Board. I am anxious to keep apart from the view of all officials in this article. My mind has been always more concentrated on the conditions of recovery, relief, or amelioration for the sick under the Poor Law than with any official questions. That these should be arranged wisely is desirable, but this is merely a question of organisation by the central authority mainly, and does not concern the far deeper and more important one of our duty to our neighbour, especially in illness.

Welcome Improvements.

Thus I come to the Order with a mind free from prejudice and only desirous of finding how this Order, if carried without very material changes, will affect the sick. And first I prefer to take the welcome improvements held out.

(1) Inmates shall not in any circumstance be employed in nursing a sick inmate. This is a gain and will, we trust, remain and be carried out in spirit and letter. I grieve that Miss Twining is not still with us to rejoice at this clear rejection of the system she thought the most dangerous part of Poor-Law nursing.

(2) *Creation of Midwives as Officials.*—Although the Midwives Act was passed in 1902, there have been great differences as to the qualifications required by Guardians for a midwife since then. I am glad to see that that useful Act will in future apply to workhouse infirmaries where a superintendent nurse is appointed *as regards the qualifications required.* Some of the rules as laid down by the Central Midwives Board are to be observed.

When I say that a slight advance on this would have been more satisfactory considering the nature of many of the maternity cases received in smaller sick wards we can only be hopeful that a midwife who is also a head nurse will be especially careful to carry out in essential matters for mother and child the duties she may have learnt." I do not, however, think it possible that any Board will appoint a midwife with "experience" only, although this Clause (5) allows them to do so. In any case the requirement for *training* as well as certification by the Central Midwives Board is generally placed in a large number of advertisements. I must now turn from my cheerful tone.

### Another Fresh Official.

A head nurse is created by the new Order as suitable for places which employ three or more nurses—one may be appointed as head nurse.

The objections to this official's requirements are (*a*) that the training as laid down is indefinite, as "a recognised school" is always difficult of recognition beyond the walls of the Local Government Board, and further (*b*) that a certificate of the Central Midwives Board is not necessary.

The head nurse will in fact take the superintendent nurse's place in future in the supervision of sick wards that may be under the 100-bed limit. She will have less complete training, we fear, and although in matters relating to the treatment and nursing of the sick she will be immediately under the orders of the medical officer, there is an uncomfortable ring about the words that the matron of the workhouse "shall supervise and control the female officers." This would in general matters apply to nurses, and we note in relation to it that one organ is pleased that the Committee "have done what they have conceived would preserve the original importance and the dignity of the position of matron."

### The Effect on the Sick.

Without altering my opinion, the position of officials one to the other should not be discussed fully here, I feel that the sick, about whom I am concerned, may suffer from an indefinite arrangement of this kind. Evidently the head nurse will not have the *power* to supervise and control the nurses under her; this is bad for the nurses and very bad for the sick. We know that sensible people constantly make better working rules than any Order can do for the harmonious working of an institution. But we must remember that human nature is not always wise, just, or able to control certain events. Every workhouse changes officials, and the ideal Order would provide for the difficulties in these small wards, and not expect too much.

The head nurse will, in fact, occupy a difficult position, and it may be one in which her best wishes cannot be carried out. This will mean more staff changes.

In relation to this it should be noted that the medical officer is not resident and cannot therefore be appealed to at once either by matron or nurse.

In fact, Articles 59 and 61 appear to me contradictory. In the first the *matron* "shall supervise and control the female officers . . . and the sick." In Article 61 the head nurse "shall superintend the other nurses and assistant nurses in the performance of their duties."

This is distinctly contradictory, and unless the articles generally are made clearer peace will not and cannot prevail.

### Small Unions.

The Committee appear to have felt unfit to frame a working policy for those institutions where the number of patients is limited. It is, however, a plain fact that in such workhouse sick wards, even if there be but one case, the inverse ratio of suffering is very severe. Take a case of arthritic rheumatism, of cancer, of heart disease, and ask if it is possible or fair to give forth only a voice of despair as to their continuous and expert nursing? I have seen these cases, perhaps four in a small Union, and I have bitterly felt the truth that the idea of limiting anything by numbers is one of the serious and cruel mistakes made.

### The Sick in Small Unions.

What is offered as a scheme for nursing in "small" Unions? The appointment of a matron who is a trained nurse, or "an arrangement" for efficient help in case of emergency or illness. Now I am well aware that in small Unions the large number of sick "go there to die;" if the disease of old age has no complications—and it often has none in agricultural districts—there still remains the acceptance by the Guardians of such serious cases as I have named above; these may require nursing for years; and it is a duty to alleviate, as only good nursing can do, the worst of their severe pain. In relation to this I would prefer to have figures to deal with: for instance, does the Local Government Board Committee mean Unions with, say, ten beds for the sick or less? My own experience has been that only in one Union were the Workhouse Nursing Association obliged to refuse to send nurses because of the actual lack of sick cases, and this was greatly regretted by the chairman and clerk who really had planned two model little sick wards for five patients, men and women. No, a bigger and a better scheme can, and I trust will, be evolved than the calling in nurses who are as a rule occupied with private nursing. Several drawbacks to this plan are (1) it is expensive, as £2 2s. is the usual charge weekly; (2) although a few nurses take such work as a change, they usually grow tired of the monotony of life in a small workhouse.

I am asked to make proposals, and I hope to write again. I think many of us who know rural workhouses well could put forward some scheme more practical and remedial than is contained in the new Order. I may hint at the usefulness of older nurses in almshouses; these are charitable institutions, whereas we are each one of us responsible for the sick, for whose efficient care we hope we are paying. If everyone in rural districts would occasionally visit their own workhouse, see and get to know its officials, the sick and the infants' nurseries, we should all soon experience the regret I for one feel at the inadequacy of "the scheme" put forward for our rural sick wards.

